# Supplementary material for: Social isolation and psychological distress among southern U.S. college students in the era of COVID-19
Source: PLoS One. 2022 Dec 30;17(12):e0279485. doi: 10.1371/journal.pone.0279485 (PMC9803292; doi:10.1371/journal.pone.0279485)
Supplement: Attachment — Submitted filename: Response to reviews.docx [file pone.0279485.s002.docx]

**JOURNAL REQUIREMENTS:**

1. When submitting your revision, we need you to address these additional requirements.

Please ensure that your manuscript meets PLOS ONE's style requirements, including those for file naming. The PLOS ONE style templates can be found at 
<https://journals.plos.org/plosone/s/file?id=wjVg/PLOSOne_formatting_sample_main_body.pdf> and 
<https://journals.plos.org/plosone/s/file?id=ba62/PLOSOne_formatting_sample_title_authors_affiliations.pdf>

**Response:** We have review the style requirements and ensured they have been incorporated into our revised manuscript.

2.  Please provide additional details regarding participant consent. In the ethics statement in the Methods and online submission information, please ensure that you have specified what type you obtained (for instance, written or verbal, and if verbal, how it was documented and witnessed). If your study included minors, state whether you obtained consent from parents or guardians. If the need for consent was waived by the ethics committee, please include this information.

**Response:** We have added that “written electronic consent was obtained from all participants” and “students had to be at least 18 years of age to be eligible to participate.”

3. Thank you for stating in your Funding Statement: 
(This research was supported by the University of North Carolina at Chapel Hill and grant F31MH119965 (PI: Giovenco) of the National Institutes of Mental Health. The funders had no role in study design, data collection and analysis, decision to publish, or preparation of the manuscript.)

Please provide an amended statement that declares *all* the funding or sources of support (whether external or internal to your organization) received during this study, as detailed online in our guide for authors at <http://journals.plos.org/plosone/s/submit-now>.  Please also include the statement “There was no additional external funding received for this study.” in your updated Funding Statement. 
Please include your amended Funding Statement within your cover letter. We will change the online submission form on your behalf.

**Response:** We have edited the funding statement: “This research was supported by the University of North Carolina at Chapel Hill and grant F31MH119965 (PI: Giovenco) of the National Institutes of Mental Health. The funders had no role in study design, data collection and analysis, decision to publish, or preparation of the manuscript. There was no additional external funding received for this study.” This has been added to the cover letter and title page.

**REVIEWER COMMENTS**

**Reviewer #1:** The manuscript addresses a timely topic and one appropriate to the journal. There are a number of strengths, including use of a sample that included a range of college student levels and provided a good bit of statistical power to work with. It is largely accessibly written (with some clarifications noted below recommended, and capable analyses was undertaken. The manuscript also has several areas where fuller elaboration and clarification would be useful. The following questions and suggestions are offered in a constructive spirit to help illuminate areas to particularly consider focus on.

**Response:** Thank you for your thorough review and constructive comments.

**Comment:** The introduction makes a range of legitimate, straightforward points. My concern is how to help readers understand early on how this paper provides complementary or value-added findings, especially coming (likely) in late 2022. There has been such a surge of reports (very much including college students) as to psychological struggles during the pandemic and the relationship of various aspects of isolation to mental health. I suggest a fuller reporting of the literature in this arena, and setting up with greater specificity how your findings extend on this.

**Response:** We agree the introduction needed to be updated. We have updated the literature in this area and formatted the last paragraph to describe specifically what our findings add.

**Comment:** Sounds like there could be a good bit of missing data if students skipping 20-25% if item responses are included. Yet, as I read the table notes at bottom (eg, Table 3), it looks like missingness was low (eg, .63% of sample). How does this square with earlier presented missingness information? It will be best to provide a clearer summary in the measurement section as to what percentage of the sample did not have their own responses for each of the psychological variables used.

**Response:** Only students who completed the survey (i.e., made it to the end of the survey even if items were missed) were included in the analysis. We have clarified this in the manuscript. Since the mental health questions were at the end of the survey, the majority of students who did not complete the survey were missing these outcomes. By removing those who partially completed the surveys, were had minimal missing data in our analytic sample. To examine the potential for bias due to excluding persons who started but did not complete the surveys, we compared the distribution of demographic characteristics, self-isolation, and psychological distress outcomes for survey completers (included in the analysis) and all survey respondents and found that the groups were similar on all measures examined. This information has been added to the supplement (S1 Table 1.1) and is also described in the statistical analysis and results sections.

**Comment:** Then these measure values went through the raking process. I understand the logic of using data from fully compliant respondents to create the measures. Yet this also raises some questions about the validity of those psych variable values for students who were not fully responsive. Did the authors consider a combination of data imputation for missingness alongside raking for sample weighting to better match the population characteristics?

**Response:** In our analysis, students with partially completed surveys are treated similarly as students who did not respond to the survey. The raking accounts for differences in demographic characteristics between those who completed the survey and those who partially completed or did not respond to the survey. In the limitations, we explain that, “although weighting methods were used to adjust for incomplete responses and nonresponse, their effectiveness is limited if there are differences between survey completers and those who partially completed or did not respond to the survey on study variables not accounted for by the weighting.”

**Comment:** Did students whose psychological variable values came from fully compliant students vary on other key variables—eg, sociodemographics? Isolating? This raises questions for interpretation of findings. At a minimum, this must be discussed in the limitations.

**Response:** We have added the following statement to the Results section: “A comparison of survey completers and all survey respondents on demographic characteristics and primary exposure and outcome variables revealed no substantive differences between groups (Supplement 1 Table 1.1).”

**Comment:** As I just now read a sentence that missing data were minimal with <2% missing doesn’t seem to add up to other missingness information presented. Please clarify this. just that just pertain to one item?
**Response:** We have edited this statement to be more specific: “Missing data for psychological distress variables in the analytic sample were minimal, with <2% of students missing data for measure items.”

**Comment:** Please provide a rationale for dichotomizing the psychological measures. Rather than using the full ranges of variance was the aim to establish equivalents of clinical cutting scores?

**Response:** We have added the statement: “When available, we dichotomized outcomes using clinically significant cutpoints to improve the interpretability of our findings.”

**Comment:** The literature has pointed out the importance of student financial resources and where they sheltered during the online period (eg, doing college from home, especially for first gen or lower income students). Not surprisingly, lower income students suffered more greatly re isolation and mental health. Students who needed to move back home with families also had a more difficult time. Possible to include these student characteristics in analysis? I strongly encourage student or family income to be used or some proxy.

**Response:** We unfortunately did not collect information on student or family income. We had added discussion of income from previous research to our Discussion section.

**Comment:** I was a little surprised to be see analysis remain at a fairly descriptive and often bivariate level. Given how much we know about isolation during COVID and its erosive associations with psychological functioning, providing a more theorized approach is likely to be useful. Why, for example, do the younger college students seem to be reporting greater struggle? Some research is using social determinant, multiple minority statuses, and/or developmental theorizing to assess how all of this affects mental health within multivariate frameworks.

**Response:** We have made substantial revisions to the Discussion to provide more interpretation of our findings and a broader description of the existing literature.

**Comment:** This richer introductory set up may then support a richer theorized discussion. The discussion notes a range of related findings and of steps that colleges/universities have been taking to reduce isolation, to increase access to supports and reduce stigma/barriers, to foster resilience and positive coping. Sharpen your message as to how your findings provide interesting extension of such points. Do you think there are ongoing shifts that will be helpful to students and student supports as we are more fully re-engaging?

**Response:** We have revised the Discussion section based on this feedback. Thank you.
